# Intriguing interfacial characteristics of the CS contact with MX_2_ (M = Mo, W; X = S, Se, Te) and MXY ((X ≠ Y) = S, Se, Te) monolayers†

**DOI:** 10.1039/d2ra00668e

**Published:** 2022-04-25

**Authors:** H. Khan, M. U. Ashraf, M. Idrees, H. U. Din, Chuong V. Nguyen, B. Amin

**Affiliations:** Department of Physics, Abbottabad University of Science & Technology Abbottabad 22010 Pakistan binukhn@gmail.com; Department of Physics, Bacha Khan University Charsadda 24420 Pakistan; Department of Materials Science and Engineering, Le Quy Don Technical University Hanoi 100000 Vietnam

## Abstract

Using (hybrid) first principles calculations, the electronic band structure, type of Schottky contact and Schottky barrier height established at the interface of the most stable stacking patterns of the CS–MX_2_ (M = Mo, W; X = S, Se, Te) and CS–MXY ((X ≠ Y) = S, Se, Te) MS vdWH are investigated. The electronic band structures of CS–MX_2_ and CS–MXY MS vdWH seem to be simple sum of CS, MX_2_ and MXY monolayers. The projected electronic properties of the CS, MX_2_ and MXY layers are well preserved in CS–MX_2_ and CS–MXY MS vdWH. Their smaller effective mass (higher carrier mobility) render promising prospects of CS–WS_2_ and CS–MoSeTe as compared to other MS vdWH in nanoelectronic and optoelectronic devices, such as a high efficiency solar cell. In addition, we found that the effective mass of holes is higher than that of electrons, suggesting that these heterostructures can be utilized for hole/electron separation. Interestingly, the MS contact led to the formation of a Schottky contact or ohmic contact, therefore we have used the Schottky Mott rule to calculate the Schottky barrier height (SBH) of CS–MX_2_ (M = Mo, W; X = S, Se, Te) and CS–MXY ((X ≠ Y) = S, Se, Te) MS vdWH. It was found that CS–MX_2_ (M = Mo, W; X = S, Se, Te) and CS–MXY ((X ≠ Y) = S, Se, Te) (in both model-I and -II) MS vdWH form p-type Schottky contacts. These p-type Schottky contacts can be considered a promising building block for high-performance photoresponsive optoelectronic devices, p-type electronics, CS-based contacts, and for high-performance electronic devices.

## Abstract 2

Electronic band structure, type of Schottky contact and Schottky barrier height established at the interface of the CS–MX_2_ (M = Mo, W; X = S, Se, Te) and CS–MXY ((X ≠ Y) = S, Se, Te) MS vdWH.

## Graphical abstract

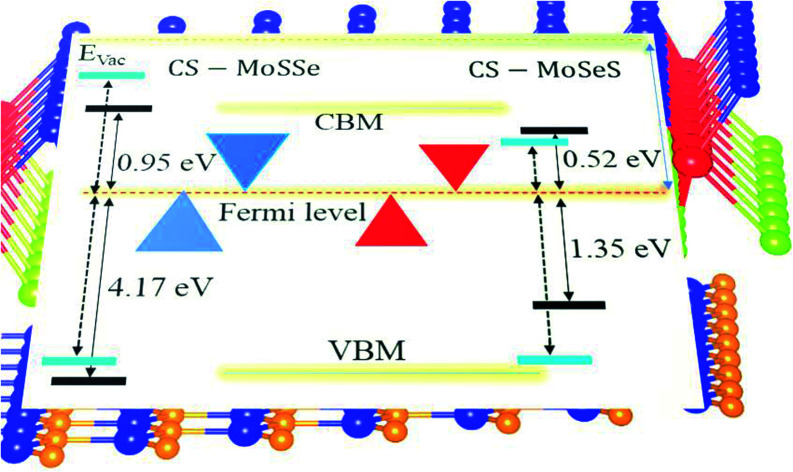

## 1. Introduction

After the successful synthesis of graphene,^1^ other two dimensional (2D) materials, such as hexagonal boron nitrides (h-BN),^2^ transition metal dichalcogenides (TMDCs),^3^ MXenes,^4^ silicene,^5^ germanene,^6^ blue and black phosphorene,^7^ borophene^8^ and stanene,^9^ have gained considerable attention in a new generation of optoelectronic and spintronic devices.^10^ In the family of 2D materials, TMDCs with MX_2_ (M = transition metal atoms, X = chalcogen atoms) stoichiometry have interesting physical/chemical properties which arise due to the structural transition from multilayers to monolayers, for example an indirect to direct bandgap transition,^11^ large exciton binding energy,^12^ and an abundance of multiexcitons.^13^ But a strong excitonic effect with high binding energies results in a very fast recombination rate of photogenerated electron and hole carriers in these materials (MX_2_ monolayers), hence leading to a low quantum efficiency.^14^ Therefore, abundant efforts have been made to tune and improve the chemical and physical properties of MX_2_ monolayers. Another class of 2D materials, XY (X = C, Si, Ge, Sn; Y= O, S, Se, Te), which exhibit planar structures^15^ with sixteen (CY, SiY, GeY and SnY; Y= O, S, Se and Te) possible combinations, consisting of an equal number of two different atoms, have attracted much attention due to their stable configuration.^16^ For each of these 2D binary monolayers, there are three different possible geometrical configurations, the puckered, buckled and planar structures. The hexagonal planar structure supports sp^2^ hybridization, whereas the favorable hybridization in group V monolayers (phosphorene and arsenene) is sp^3^, which shows that the hybridization in group IV–VI binary monolayers is similar to those of phosphorene and arsenene. It is observed that CS monolayers in the planar configuration are metallic due to the strong overlap of the conduction and valence bands.^15^

Lu (Zhang) *et al.*^17^(^18^) have selenized (sulfurized) MoS_2_(MoSe_2_) through a chemical vapor deposition (CVD) technique and named these Janus transition metal dichalcogenides (JTMDCs) with the chemical formula MXY (M = Mo, W; (X ≠ Y) = S, Se). These materials have been shown to be promising for spintronic devices due to the SOC-induced Rashba spin splitting.^19^ Using density functional theory (DFT) calculations, Xia *et al.*^20^ showed that the atomic radius and electronegativity differences of the X and Y chalcogen atoms in MXY (M = Mo, W; X, Y = S, Se, Te) monolayers are associated with the direct to indirect bandgap transition and induced dipole moment. Furthermore, Idrees *et al.*^21^ have also used DFT and shown that MoSSe, WSSe, MoSeTe and WSeTe (MoSTe and WSTe) monolayers are direct (indirect) bandgap semiconductors. They transformed indirect MoSTe and WSTe to direct bandgap semiconductors by using external electric fields. They have also investigated the absorption spectra, absorption efficiency, and photocatalytic behavior of these materials.

The stacking of isolated 2D materials *via* van der Waals forces in a precisely controlled sequence produces van der Waals heterostructures (vdWH).^22^ This provides a versatile platform for exploring the uses of new phenomena in designing novel nanoelectronic devices.^23,24^ In this regard, the stackings of semiconductors with semiconductors (SS contact) and metals with semiconductors (MS contact) are of crucial importance, with a wide range of device applications.^25^ To date, many of the vdWH in the form of SS contacts have been investigated both theoretically^26–37^ and experimentally^38–41^ for novel extraordinary applications in optoelectronic devices.^42–47^

In the case of MS contacts, the Schottky barrier (SB) is an energy barrier across the junction for the transport of carriers.^48^ It reduces the contact resistance, modulates carrier polarity in the channel for transistors, and also enhances the selectivity of carrier extraction for photovoltaic cells,^49,50^ hence it plays a key role in device performance. In MS contacts, there is another important phenomena, the Fermi level pinning (FLP) caused by metal-induced gap states (MIGS) and interface dipoles or defects created at the interface.^51^ It refers to the insensitivity of the SB to the work function of the metal.^52^ TMDCs have been used in almost every MS contact in both experiments^53,54^ and theory.^55,56^ The contact of single layer MoS_2_ (semiconductor) has already been proposed with Ti (metal)^57^ and other metals of varying work functions.^58^

Indeed, the small lattice mismatch and identical symmetry of CS, MX_2_ (M = Mo, W; X = S, Se, Te) and MXY ((X ≠ Y) = S, Se, Te) monolayers allow the creation of MS contacts in the form of CS–MX_2_ and CS–MXY vdWH. Alternative ordering of the chalcogen atoms allows the creation of two models of the CS–MXY vdWH. Therefore, we have fabricated the possible stacking patterns in CS–MX_2_ and in both (two) models of CS–MXY MS vdWH. After making the possible stacking configurations, we have investigated the electronic band structure, type of Schottky contact and Schottky barrier height established at the interface of the most stable stacking patterns of the MS vdWH under investigation. These findings show the capability to control and modify the properties of the CS, MX_2_ (M = Mo, W; X = S, Se, Te) and MXY ((X ≠ Y) = S, Se, Te) monolayers, and provide guidelines for the designing of high-performance devices based on MS vdWH.

## 2. Computational details

We have used DFT^59^ with the empirical dispersion correction of Grimme,^60^ and the functionals of Perdew–Burke–Ernzerhof (PBE)^61^ and Heyd–Scuseria–Ernzerhof (HSE06)^62^ in the Vienna *ab initio* simulation package (VASP).^63,64^

*Γ*-point centered 6 × 6 × 1 Monkhorst–Pack *k*-point grids in the first Brillouin zone and a cutoff energy of 500 eV were used in the PBE functionals for the geometric relaxations until achieving the convergence criterion of 10^−4^ eV Å^−1^ (10^−5^ eV) for forces (energy). The Monkhorst–Pack *k*-point grids were refined to 12 × 12 × 1 for the electronic structure calculations. The converged PBE wave functions were further used for HSE06 calculations, while the *k*-mesh here was not refined due to the high computational costs. A 25 Å vacuum layer thickness was used to avoid interactions between adjacent layers.

We have also performed *ab initio* molecular dynamics (AIMD) simulations,^65^ through the Nose thermostat algorithm at a temperature of 300 K for a total of 6 ps with a time interval of 1 fs to investigate the thermal stabilities of CS–MX_2_ (M = Mo, W; X = S, Se, Te) and CS–MXY ((X ≠ Y) = S, Se, Te) MS vdWH.

Using the Quantum ESPRESSO package, the Bethe–Salpeter equation (BSE) was also solved using the GW method^66^ to investigate the optical spectra of the imaginary part of the dielectric functions (*ε*_2_(*ω*)) of CS, the MX_2_ (M = Mo, W; X = S, Se, Te) and MXY ((X ≠ Y) = S, Se, Te) monolayers and the CS–MX_2_ (M = Mo, W; X = S, Se, Te) and CS–MXY ((X ≠ Y) = S, Se, Te) MS vdWH.^67–69^

## 3. Results and discussion

The calculated lattice parameters (lattice constant, bond length), electronic structure (bandgap values), and the photocatalytic and optical response of the CS (see Fig. S1†), MX_2_ (M = Mo, W; X = S, Se, Te), and MXY ((X ≠ Y) = S, Se, Te) monolayers are found to be in agreement with ref. 15 and 18–21, hence showing the validity of the same approach for the calculations of the CS–MX_2_ (M = Mo, W; X = S, Se, Te) and CS–MXY ((X ≠ Y) = S, Se, Te) MS vdWH.

**Fig. 1:**
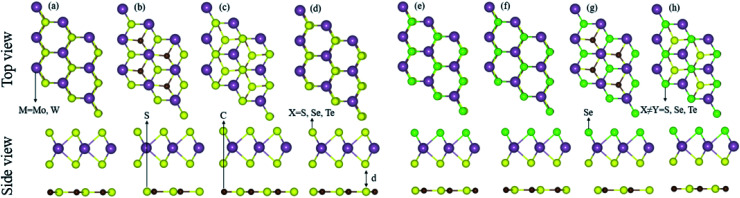
Stacking configurations of the CS–MX_2_ (X = S, Se, Te) (a)–(d) and CS–MXY ((X ≠ Y) = S, Se, Te) MS vdWH in model-I (e)–(h), see the text for details.

The lattice mismatch of CS with MX_2_ (1–11%), and with MXY (2–7%) monolayers is experimentally achievable^70^ in the fabrication of CS–MX_2_ and CS–MXY MS vdWH. Furthermore, the same hexagonal symmetry of the CS monolayer, as shown in Fig. S1,† and the MX_2_ (M = Mo, W; X = S, Se, Te) and MXY ((X ≠ Y) = S, Se, Te) monolayers also allows the formation of these MS vdWH. The electronic band structure and stability of vdWH are very sensitive to layer stacking,^71^ therefore, four possible stacking patterns for the CS–MX_2_ (M = Mo, W; X = S, Se, Te) and CS–MXY ((X ≠ Y) = S, Se, Te) MS vdWH are fabricated, see Fig. 1. In the case of the CS–MX_2_ (M = Mo, W; X = S, Se, Te) MS vdWH (Fig. 1(a)–(d)): in stacking (a), the M(X) atom of the MX_2_ layer is placed on top of the S(C) atom of the CS layer, in stacking (b), the M atom of the MX_2_ layer is placed on the top of the S atom of the CS layer, while both X atoms of the MX_2_ layer and the C atom of the CS layer are on the hexagonal site, in stacking (c), the M atom of the MX_2_ layer and S atoms of the CS layer are placed on hexagonal sites, while the X atoms of the MX_2_ layer is placed on top of the C atom of the CS layer, and in stacking (d), the M(X) atom of the MX_2_ layer is placed on the top of the C(S) atom of the CS layer. In the case of the CS–MXY ((X ≠ Y) = S, Se, Te) MS vdWH, two different chalcogen atoms (X and Y) are attached to the transition metal atom (M), therefore eight possible high-symmetry stacking sequences of layers are fabricated, separated into two models, with each having four stacking patterns. In model-I, similar chalcogen atoms are placed at the interface of two layers *i.e.* CS–MXY, see Fig. 1((e)–(h), while in model-II, different chalcogen atoms are placed at the interface of the two layers, *i.e.* CS–MYX, see Fig. S2(a)–(d).† In model-I of the CS–MXY ((X ≠ Y) = S, Se, Te) vdWH: in stacking (e), the M(X,Y) atom of the MXY layer is placed on top of the C(S) atom of the CS layer, in stacking (f), the M(X,Y) atom of the MXY layer is placed on top of the S(C) atom of the CS layer, in stacking (g), the M atom of the MXY layer is placed on top of the S atom of the CS layer, while both the X and Y atoms of the MXY layer and the C atom of CS layer are placed on hexagonal sites, and in stacking (h), the M atom of the MXY layer is placed on a hexagonal site, while both the X and Y atoms of the MXY layer are placed on top of the C atom of the CS layer. We have also evaluated the similar stacking patterns in model-II of the CS–MXY ((X ≠ Y) = S, Se, Te) vdWH with an alternative order of the chalcogen atoms, see Fig. S2(a)–(d).†

The binding energies, *E*_b_ = *E*_(CS–MX2(CS–MXY))_ − *E*_(CS)_ − *E*_(MX2(MXY))_, where *E*_(CS–MX2(CS–MXY))_ is the total energy of the CS–MX_2_(CS–MXY) MS vdWH, *E*_(CS)_ is the total energy of the isolated CS monolayer and *E*_(MX2(MXY))_ is the total energy of the isolated MX_2_(MXY) monolayer, and the interlayer distances are presented in Table 1. Smaller interlayer distances and binding energies represent the thermodynamically most stable stacking pattern, therefore, stacking (b) of the CS–MX_2_ (M = Mo, W; X = S, Se, Te) and stacking (d) of model-I of the CS–MXY ((X ≠ Y) = S, Se, Te) MS vdWH are the thermodynamically most stable stacking patterns. In the case of model-II of the CS–MXY ((X ≠ Y) = S, Se, Te) MS vdWH, stacking (b) for CS–MoSeS, CS–MoTeS and CS–WTeSe, and stacking (c) for CS–WSeS, CS–WTeS and CS–MoTeSe vdWH were found to be the thermodynamically most stable stacking patterns. The varying stable stacking in the case of model-II of the CS–MXY ((X ≠ Y) = S, Se, Te) MS vdWH is due to the induced strain on account of the different chalcogen atoms and also may be due to the unlike interface atoms compared to model-I. These thermodynamically most stable stacking patterns of the MS vdWH under investigation are considered for further investigations. The negative binding energies show that the formation of all MS vdWHs is exothermic, see Table 1, hence recommending the experimental fabrication of the CS–MX_2_ and CS–MXY MS vdWH. These values are in the range of the binding energies for other vdWHs.^21,72,73^ The calculated interlayer distances (see Table 1) also confirm weak vdW interactions in the stacked layers of the MS vdWHs under investigation. The optimized lattice constants and bond length of the CS–MX_2_ (M = Mo, W; X = S, Se, Te) and CS–MXY ((X ≠ Y) = S, Se, Te) MS vdWH are presented in Table 2.

Binding energies (eV) and interlayer distances (Å) of the possible configuration of the CS–MX_2_ (M = Mo, W; X = S, Se, Te) and CS–MXY (M = Mo, W; (X ≠ Y) = S, Se, Te) MS vdWH

**Table:** 

CS–MX_2_		CS–MoS_2_	CS–MoSe_2_	CS–MoTe_2_	CS–WS_2_	CS–WSe_2_	CS–WTe_2_
Stacking (a)	*E*_b_ (eV)	−0.44	−0.36	−0.79	−0.18	−0.60	−1.22
	*d*	3.63	3.43	3.54	3.42	3.43	3.54
Stacking (b)	*E*_b_ (eV)	−0.50	−0.62	−0.93	−0.55	−1.03	−1.22
	*d*	3.41	3.43	3.42	3.42	3.41	3.47
Stacking (c)	*E*_b_ (eV)	−0.49	−0.13	−0.86	−0.51	−0.47	−0.95
	*d*	3.41	3.43	3.51	3.51	3.43	3.47
Stacking (d)	*E*_b_ (eV)	−0.46	−0.58	−0.87	−0.51	−0.83	−0.89
	*d*	3.41	3.43	3.54	3.42	3.43	3.47

**Table:** 

CS–MXY (model-I)		CS–MoSSe	CS–MoSTe	CS–MoSeTe	CS–WSSe	CS–WSTe	CS–WSeTe
Stacking (e)	*E*_b_ (eV)	−0.38	−0.10	−0.11	−0.43	−0.14	−0.14
	*d*	3.42	3.44	3.44	3.42	3.45	3.45
Stacking (f)	*E*_b_ (eV)	−0.11	−0.11	−0.13	−0.41	−0.15	−0.16
	*d*	3.42	3.42	3.43	3.40	3.42	3.43
Stacking (g)	*E*_b_ (eV)	−0.47	−0.11	−0.20	−0.38	−0.15	−0.21
	*d*	3.42	3.41	3.41	3.42	3.45	3.45
Stacking (h)	*E*_b_ (eV)	−0.51	−0.17	−0.69	−0.53	−0.22	−0.76
	*d*	3.40	3.41	3.41	3.40	3.42	3.43

**Table:** 

CS–MXY (model-II)		CS–MoSeS	CS–MoTeS	CS–MoTeSe	CS–WSeS	CS–WTeS	CS–WTeSe
Stacking (a)	*E*_b_ (eV)	−0.43	−0.61	−0.63	−0.73	−1.53	−0.73
	*d*	3.42	3.42	3.44	3.42	3.43	3.45
Stacking (b)	*E*_b_ (eV)	−0.48	−0.69	−0.71	−0.03	−0.66	−0.95
	*d*	3.40	3.41	3.43	3.42	3.44	3.42
Stacking (c)	*E*_b_ (eV)	−0.19	−0.39	−0.74	−0.74	−1.56	−0.25
	*d*	3.42	3.42	3.41	3.40	3.42	3.45
Stacking (d)	*E*_b_ (eV)	−0.39	−0.39	−0.42	−0.03	−0.66	−0.44
	*d*	3.43	3.43	3.42	3.40	3.42	3.43

Lattice constants (*a* in Å), bond lengths (in Å), work functions (*ϕ* in eV) and potentials (Δ*V* in eV) of the CS–MX_2_ (M = Mo, W; X = S, Se, Te) and CS–MXY ((X ≠ Y) = S, Se,Te) MS vdWH

**Table:** 

CS–MX_2_	CS–MoS_2_	CS–MoSe_2_	CS–MoTe_2_	CS–WS_2_	CS–WSe_2_	CS–WTe_2_
*a*	3.18	3.25	3.59	3.20	3.25	3.37
M–X	2.48	2.51	2.66	2.49	2.51	2.56
C–S	1.83	1.88	2.06	1.85	1.88	1.85
*ϕ*	1.90	1.53	2.22	1.92	1.78	1.46
Δ*V*	−10.37	−2.53	−0.76	−2.03	−1.16	−5.30
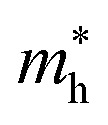	0.0076	0.0061	0.0089	0.0051	0.0071	0.0057
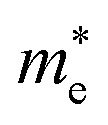	0.0045	0.0042	−0.0064	0.0034	0.0052	0.0042

**Table:** 

CS–MXY (model-I)	CS–MoSSe	CS–MoSTe	CS–MoSeTe	CS–WSSe	CS–WSTe	CS–WSeTe
*a*	3.21	3.26	3.29	3.21	3.27	3.31
M–X	2.42	2.71	2.70	2.42	2.71	2.70
M–Y	2.41	2.40	2.51	2.41	2.41	2.52
C–S	1.75	1.78	1.78	2.07	1.79	1.80
*ϕ*	2.15	2.71	2.67	1.90	2.55	2.67
Δ*V*	−9.61	−9.86	−12.15	−9.35	−9.61	−12.28
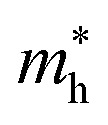	0.0061	0.0055	0.0049	0.0068	0.0045	0.0051
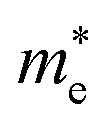	0.0047	0.0040	0.0037	0.0043	0.0031	0.0034

**Table:** 

CS–MXY (model-II)	CS–MoSSe	CS–MoSTe	CS–MoSeTe	CS–WSSe	CS–WSTe	CS–WSeTe
*a*	3.20	3.25	3.25	3.21	3.28	3.30
M–X	2.52	2.40	2.51	2.48	2.51	2.53
M–Y	2.41	2.72	2.51	2.49	2.52	2.53
C–S	1.85	2.37	1.88	1.67	1.70	1.82
*ϕ*	1.64	2.02	2.15	1.89	1.38	2.18
Δ*V*	−9.14	−10.08	−8.62	−7.33	−2.28	−7.80
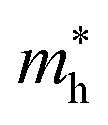	0.0053	0.0061	0.0050	0.0073	0.0071	0.0052
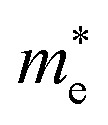	0.0029	0.0035	0.0042	0.0029	0.0060	0.0038

**Fig. 2:**
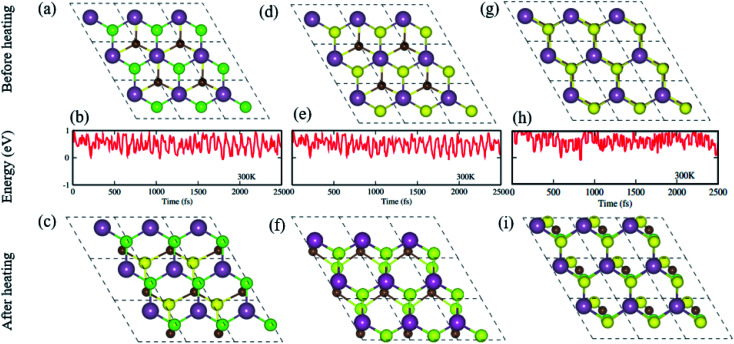
Geometrical structures before heating (first row), with fluctuating energy (second row) and after heating (third row) of the CS–MoS_2_ (a)–(c) and CS–MoSSe in model-I (d)–(f) and in model-II (g)–(i) MS vdWH using AIMD simulations.

Furthermore, we have performed AIMD simulations^74,75^ to verify the thermal stability of the MS vdWHs under investigation. There is no structural distortion in the CS–MX_2_ (M = Mo, W; X = S, Se, Te) and CS–MXY ((X ≠ Y) = S, Se, Te) vdWH after heating them for 6 ps. The fluctuation in the total energy at 0 ps and 6 ps is very small, indicating that these configuration are thermally stable at 300 K, making these systems feasible and they can be obtained easily in future experiments.^70^ From AIMD simulations, the geometrical structures before heating (first row), with fluctuating energy (second row) and after heating (third row) of CS–MoS_2_, and CS–MoSSe in both model-I and -II MS vdWH are presented in Fig. 2.

**Fig. 3:**
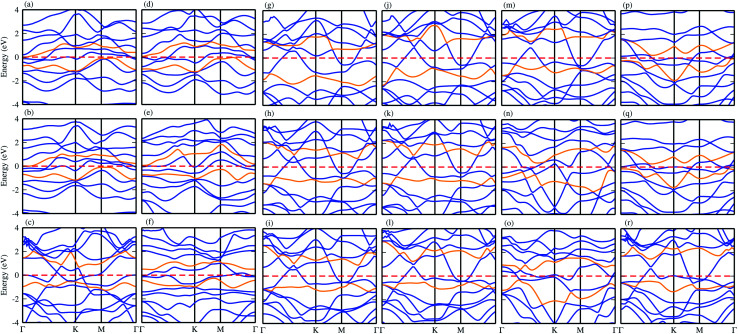
Band structures of the (a) CS–MoS_2_, (b) CS–MoSe_2_, (c) CS–MoTe_2_, (d) CS–WS_2_, (e) CS–WSe_2_, (f) CS–WTe_2_, (g) CS–MoSSe, (h) CS–MoSTe (i) CS–MoSeTe, (j) CS–WSSe, (k) CS–WSTe, (l) CS–WSeTe, (m) CS–MoSeS (n) CS–MoTeS (o) CS–MoTeSe (p) CS–WSeS, (q) CS–WTeS and (r) CS–WTeSe MS vdWH using PBE functionals.

Using the PBE functional, the calculated electronic band structures of CS–MX_2_ and CS–MXY in model-I and -II MS vdWH are calculated and are presented in Fig. 3. It has been shown in ref. 15 that the CS monolayer has zero bandgap with indirect Dirac cones at the *Γ*–*K* and *M*-points of the BZ, while the MX_2_ (M = Mo, W; X = S, Se, Te) monolayers are direct bandgap semiconductors with the CBM (VBM) lying at the *K* point of the first BZ.^76^ Similarly, in MXY (M = Mo, W; (X ≠ Y) = S, Se, Te) monolayers, MoSSe, WSSe, MoSeTe and WSeTe are direct bandgap semiconductors, while MoSTe and WSTe are *Γ*–*K*-point indirect bandgap semiconductors.^21^ The electronic band structures of the CS–MX_2_(CS–MXY) MS vdWH seem to be simple sums of the CS and MX_2_(MXY) monolayers, see Fig. 3. The Dirac like cone of the CS layer (the same as graphene) is also present in the CS–MX_2_(CS–MXY) MS vdWH. Most interestingly, we notice that the CS layer has opened a tiny bandgap after stacking with MX_2_ and MXY layers in the form of the MS vdWH, which is comparable with graphene based vdWH, such as G-MoS_2_,^77^ G-GeTe,^78^ G-GeC^79^ and G-SnO.^80^ The opening of the bandgap of CS monolayer is due to the lattice symmetry breaking while making the CS–MX_2_ (M = Mo, W; X = S, Se, Te) and CS–MXY ((X ≠ Y) = S, Se, Te) MS vdWH. All these results demonstrate that the projected electronic properties of the CS, MX_2_ and MXY layers are well preserved in the CS–MX_2_ and CS–MXY MS vdWH.

**Fig. 4:**
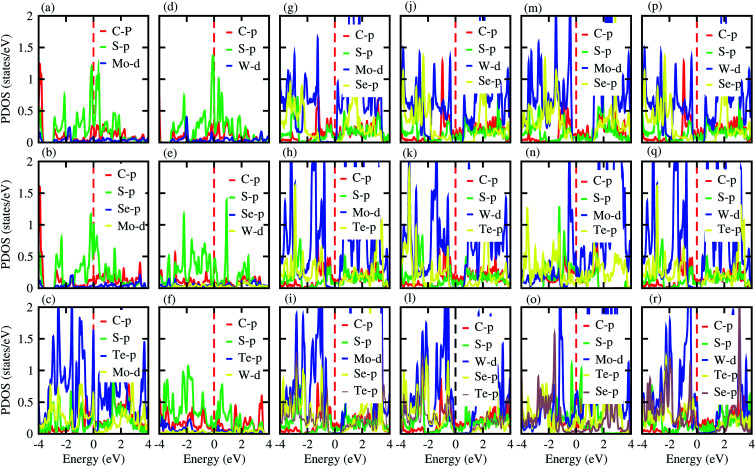
PDOS of the (a) CS–MoS_2_, (b) CS–MoSe_2_, (c) CS–MoTe_2_, (d) CS–WS_2_, (e) CS–WSe_2_, (f) CS–WTe_2_, (g) CS–MoSSe, (h) CS–MoSTe, (i) CS–MoSeTe, (j) CS–WSSe, (k) CS–WSTe, (l) CS–WSeTe, (m) CS–MoSeS, (n) CS–MTeS, (o) CS–MoTeSe, (p) CS–WSeS, (q) CS–WTeS and (r) CS–WTeSe MS vdWH using PBE functionals.

Furthermore, contributions of the orbitals of the CS and MX_2_(MXY) monolayers in the corresponding CS–MX_2_ and CS–MXY in model-I and -II MS vdWH are investigated by partial density of states (PDOS), see Fig. 4. One can see that in the PDOS, by making the CS–MX_2_ and CS–MXY vdWH, the CBM of the MX_2_ and MXY layers are shifted towards the Fermi level, which is due to the stacking on the CS monolayer, while the main contributions are due to the C-p and S-p orbitals of the CS monolayers (which cross the Fermi level) in the CS–MX_2_ and CS–MXY MS vdWH, respectively. An approach in DFT, that hybrid functionals lead to better agreement with experiments than semi-local functionals, is not general,^81^ but depends on the considered materials. Therefore, we have also used the HSE06 functional to investigate the electronic band structures of the CS–MX_2_ (M = Mo, W; X = S, Se, Te) and CS–MXY ((X ≠ Y) = S, Se, Te) vdWH, see Fig. S3.† Using the HSE06 functional, these MS vdWH show similar band structures to the PBE functionals with a small shift in the CBM towards a higher energy.

**Fig. 5:**
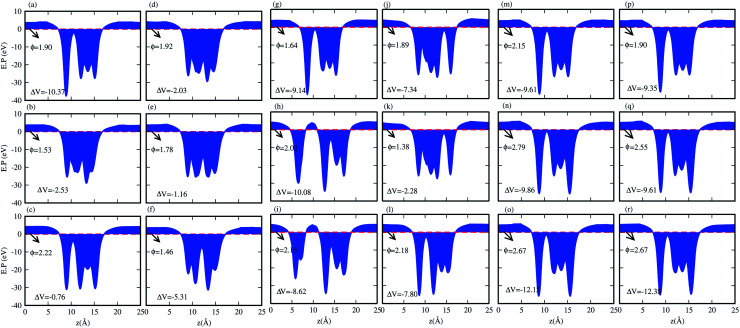
Average electrostatic potentials of the (a) CS–MoS_2_, (b) CS–MoSe_2_, (c) CS–MoTe_2_, (d) CS–WS_2_, (e) CS–WSe_2_, (f) CS–WTe_2_, (g) CS–MoSSe, (h) CS–MoSTe (i) CS–MoSeTe, (j) CS–WSSe, (k) CS–WSTe, (l) CS–WSeTe, (m) CS–MoSeS (n) CS–MoTeS (o) CS–MoTeSe (p) CS–WSeS, (q) CS–WTeS and (r) CS–WTeSe MS vdWH using PBE functionals.

We have also calculated the electrostatic potentials of the CS–MX_2_ (M = Mo, W; X = S, Se, Te) and CS–MXY ((X ≠ Y) = S, Se, Te) in model-I and -II MS vdWH, see Fig. 5. The electrostatic potential difference (Δ*V*), presented in Table 2, lies in the range of −0.76 to −12.28 eV. The MX_2_(MXY) monolayers have deeper electrostatic potentials than that of the CS monolayer in CS–MX_2_(CS–MXY) MS vdWH. This difference in the electrostatic potentials may have a crucial impact on the charge injection and carrier dynamics when these systems are used as electrodes.^82^ It should be noted that a large potential difference will significantly influence the charge transportation of the 2D MS vdWH. This electrostatic potential at the interface of CS–MX_2_ and CS–MXY MS vdWH can successfully reduce the charge carrier recombination and increase the transfer and separation of the induced charge carriers, which enhances the power conversion efficiency.^83^ The surface conditions of the material affect the work function due to altering the surface electric field induced by the distribution of electrons at the interface.^84^ The calculated values of the work functions for the CS–MX_2_ and CS–MXY MS vdWH lie in the range of 1.46 to 2.71 eV, see Tables 2 and S1,† which show a good response for field effect transistors (FETs).^85^ Using the HSE06 functional, the calculated average electrostatic potential of the CS–MX_2_ (M = Mo, W; X = S, Se, Te) and CS–MXY ((X ≠ Y) = S, Se, Te) in model-I and -II MS vdWH are presented in Fig. S4 and Table S1.†

Charge redistribution and transfer (quantitatively) from one layer to the other layer are investigated by charge density difference and Bader charge analysis using Δ*ρ* = *ρ*_(CS–MX2(CS–MXY))_ − *ρ*_(CS)_ − *ρ*_(MX2(MXY))_, where Δ*ρ* is the total charge density difference, *ρ*_(CS–MX2(CS–MXY))_ is the charge density of the CS–MX_2_(CS–MXY) vdWH, *ρ*_(CS)_ is the charge density of the CS monolayer, and *ρ*_(MX2(MXY))_ is the charge density of the MX_2_ or MXY monolayer. In the case of the CS–MX_2_ vdWH, about 0.0023, 0.005, 0.429, 0.0052, 0.175, and 0.0806 electrons are transferred from the CS to the MoS_2_, MoSe_2_, MoTe_2_, WS_2_, WSe_2_, and WTe_2_ layer, respectively, at the interface of the CS–MX_2_ (M = Mo, W; X = S, Se, Te) MS vdWH. Similarly, in the case of the CS–MXY ((X ≠ Y) = S, Se, Te) MS vdWH in model-I (-II), about 0.0106(0.0351), 0.005(0.0135), 0.0044(0.0089), 0.0038(0.0293), 0.004(0.0091), and 0.005 (0.029) electrons are transferred from the CS to the MoSSe, MoSTe, MoSeTe, WSSe, WSTe, and WSeTe layers at the interface, respectively.

The effective mass of the CS–MX_2_ (M = Mo, W; X = S, Se, Te) and CS–MXY ((X ≠ Y) = S, Se, Te) MS vdWH are calculated by using 
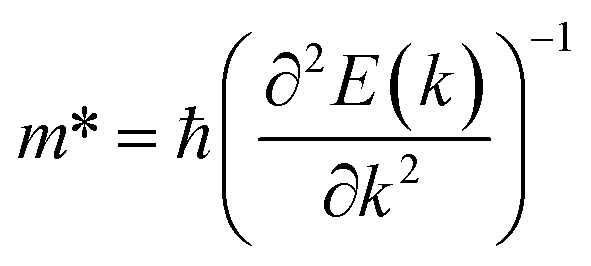
 (ref. 86) and are presented in Table 2. The smaller values of the effective mass (for holes and electrons) indicate that the CS–MX_2_ and CS–MXY MS vdWH have high carrier mobility *i.e.*
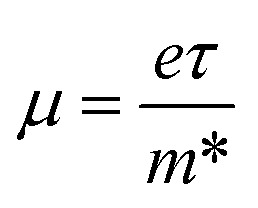
 and, hence, are suitable for high performance nanoelectronic devices. From Table 2, one can see that CS–WS_2_ and CS–MoSeTe have smaller effective mass (higher carrier mobility) as compared to those of the other vdWH, demonstrating that these heterostructures render promising prospects for nanoelectronic and optoelectronic devices, such as a high efficiency solar cell. In addition, we found that the effective mass of holes is higher than that of electrons, suggesting that these heterostructures can be utilized for hole/electron separation.^87^ Using the HSE06 functional, the calculated carrier effective mass of the CS–MX_2_ (M = Mo, W; X = S, Se, Te) and CS–MXY ((X ≠ Y) = S, Se, Te) in model-I and -II MS vdWH are presented in Table S1.†

**Fig. 6:**
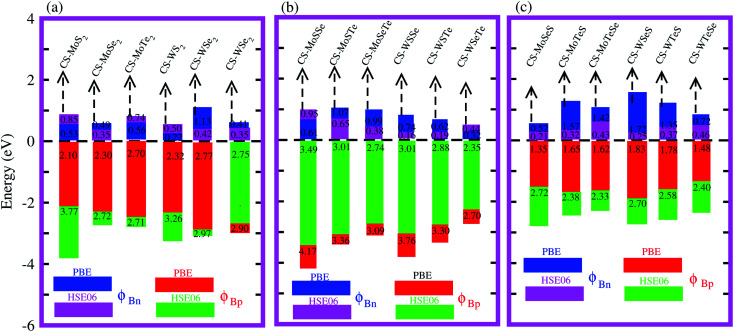
Calculated Schottky barrier values for the CS–MX_2_ (M = Mo, W; X = S, Se, Te) (a) and CS–MXY ((X ≠ Y) = S, Se, Te) in model-I (b) and in model-II (c) MS vdWH.

**Fig. 7:**
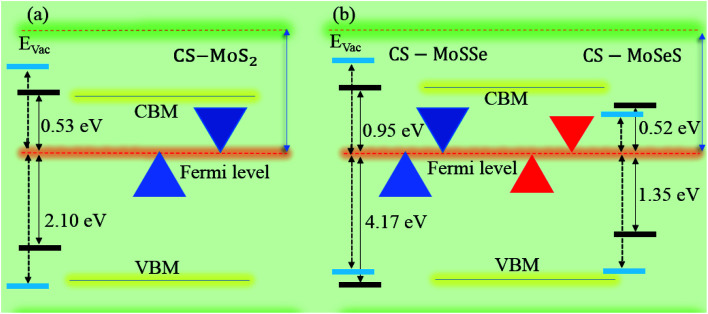
Band alignment of the (a) CS–MoS_2_ and (b) CS–MoSSe and CS–MoSeS MS vdWH. The dotted lines represent the HSE06 calculations.

Interestingly, MS contact led to the formation of a Schottky contact or ohmic contact. We can see from the electronic band structures in Fig. 3 and S2† that the Fermi levels of the CS–MX_2_(CS–MXY) MS vdWH lie between the CBM and VBM of the MX_2_ (M = Mo, W; X = S, Se, Te) and MXY ((X ≠ Y) = S, Se, Te) monolayers, thus forming a Schottky contact. Using the Schottky Mott rule,^88^ the Schottky barrier height (SBH) of n(p) type Schottky contacts is calculated as *Φ*_B,n_ = *E*_CBM_ − *E*_F_(*Φ*_B,p_ = *E*_F_ − *E*_CBM_), and the computed values of *Φ*_B,n_(*Φ*_B,p_)^48^ are presented in Fig. 6. One can see that *Φ*_B,p_ have higher values than *Φ*_B,n_, thus, the CS–MX_2_ (M = Mo, W; X = S, Se, Te) and CS–MXY ((X ≠ Y) = S, Se, Te) (in both model-I and -II) vdWH form p-type Schottky contacts. These p-type Schottky contacts can be considered to be a promising building block for high-performance photoresponsive optoelectronic devices,^89^ p-type electronics,^90^ CS–based contacts,^91^ and for high-performance electronic devices.^92^ While making the CS–MX_2_ (M = Mo, W; X = S, Se, Te) and CS–MXY ((X ≠ Y) = S, Se, Te) vdWH, there is no chemical bond among CS and MX_2_ (MXY) layers, which may create an interface dipole, which can be calculated *via* the potential step Δ*ρ*, as presented in Fig. 7. In the case of the SBH of p(n)-type, *Φ*_B,n_ = *W*_CS_ + Δ*V* − *χ*_(CS–MX2,CS–MXY)_ (*Φ*_B,n_ = *I*_(CS–MX2,CS–MXY)_ − *W*_CS_ + Δ*V*), where *W* represents the calculated work function *χ* is the electron affinity and *I* is the ionization energies of the vdWH and corresponding monolayers. We have calculated the work function and Δ*V*, presented in Table 2. The calculated values of *Φ*_B,n_ and *Φ*_B,p_ with and without considering Δ*V* are quite unchanged. Hence, the interface dipole at the CS–MX_2_ and CS–MXY vdWH is neglected within the vdW layers.^93^

**Fig. 8:**
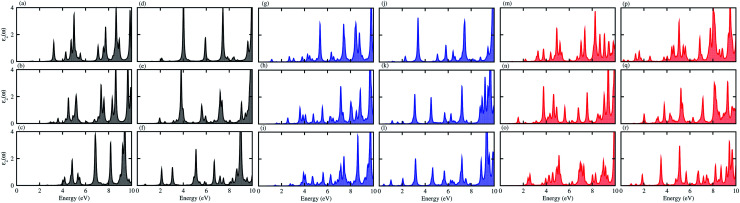
*ε*_2_(*ω*) of the (a) CS–MoS_2_, (b) CS–MoSe_2_, (c) CS–MoTe_2_, (d) CS–WS_2_, (e) CS–WSe_2_, (f) CS–WTe_2_, (g) CS–MoSSe, (h) CS–MoSTe, (i) CS–MoSeTe, (j) CS–WSSe, (k) CS–WSTe, (l) CS–WSeTe, (m) CS–MoSeS, (n) CS–MTeS, (o) CS–MoTeSe, (p) CS–WSeS, (q) CS–WTeS and (r) CS–WTeSe MS vdWH.

For use in practical applications in optoelectronic and photocatalytic nano devices, we have further calculated the imaginary parts of the dielectric function (*ε*_2_(*ω*)) of the CS, MX_2_ (M = Mo, W; X = S, Se, Te) and MXY ((X ≠ Y) = S, Se, Te) monolayers, see Fig. S4† and the CS–MX_2_ and CS–MXY ((in both model-I and -II) MS vdWH, see Fig. 8. One can see that the *ε*_2_(*ω*) spectra of the CS, MX_2_ (M = Mo, W; X = S, Se, Te) and MXY ((X ≠ Y) = S, Se, Te) monolayers, (see Fig. S5†) and the CS–MX_2_ and CS–MXY ((in both model-I and -II) MS vdWH (see Fig. 8) exhibit an intense absorption peak near the visible region, which suggests the visible light absorption capability of these systems. Fig. S4† also shows that the *ε*_2_(*ω*) spectrum of CS is very weak as compared to those of TMDCs and JTMDCs. Furthermore, a slight blueshift is found in the spectra of all MS vdWH compared to those of the isolated monolayers. Fig. 8 also shows that the absorption intensity of the *ε*_2_(*ω*) spectra for the vdW heterostructures overlaps with those of TMDCs and JTMDCs but is higher than that of the CS monolayer. This indicates the good absorption capability of the constructed heterostructure.^94^

## 4. Conclusion

Lattice mismatch and the same hexagonal symmetry of the CS (metal) and the MX_2_ (M = Mo, W; X = S, Se, Te) and MXY ((X ≠ Y) = S, Se, Te) (semiconductor) monolayers also allow the formation of MS contacts in the form of vdWH. Therefore, using (hybrid) first principles calculations, we have investigated the electronic band structure, type of Schottky contact and Schottky barrier height established at the interface of the most stable stacking patterns of the CS–MX_2_ (M = Mo, W; X= S, Se, Te) and CS–MXY ((X ≠ Y) = S, Se, Te) MS vdWH. The calculated electronic band structures show that the projected electronic properties of the CS, MX_2_ and MXY monolayers are well preserved in the CS–MX_2_ and CS–MXY MS vdWH. The smaller effective mass (higher carrier mobility) of electrons and holes render promising prospects of CS–WS_2_ and CS–MoSeTe as compared to other MS vdWH in nanoelectronic and optoelectronic devices. Interestingly, the MS contact of the CS (metal) and MX_2_ (M = Mo, W; X = S, Se, Te), and MXY ((X ≠ Y) = S, Se, Te) (semiconductors) monolayers led to the formation of a Schottky contact or ohmic contact, therefore we have used the Schottky Mott rule to calculate the Schottky barrier height (SBH) of the CS–MX_2_ (M = Mo, W; X = S, Se, Te) and CS–MXY ((X ≠ Y) = S, Se, Te) MS vdWH. The CS–MX_2_ (M = Mo, W; X = S, Se, Te) and CS–MXY ((X ≠ Y) = S, Se, Te) (in both model-I and -II) MS vdWH form p-type Schottky contacts, a promising building block for high-performance photoresponsive optoelectronic devices, p-type electronics, CS-based contacts, and for high-performance electronic devices.

## Conflicts of interest

There are no conflicts to declare.

## Supplementary Material

RA-012-D2RA00668E-s001
