# Supplementary material for: Intriguing interfacial characteristics of the CS contact with MX2 (M = Mo, W; X = S, Se, Te) and MXY ((X ≠ Y) = S, Se, Te) monolayers
Source: RSC Adv. 2022 Apr 25;12(20):12292–302. doi: 10.1039/d2ra00668e (PMC9036409; doi:10.1039/d2ra00668e)
Supplement: RA-012-D2RA00668E-s001 [file RA-012-D2RA00668E-s001.pdf]

Electronic Supplementary Material (ESI) for RSC Advances.
This journal is © The Royal Society of Chemistry 2022

Intriguing Interfacial Characteristics of CS Contact to MX_2 (M=Mo, W; X=S, Se, Te) and MXY ((X≠Y) = S, Se, Te)) Monolayers

H. Khan[1], M. U. Ashraf[1], M. Idrees[1], H. U. Din[2], Chuong V. Nguyen[3], B. Amin[1*]

[1]*Department of Physics, Abbottabad University of Science & Technology, Abbottabad 22010, Pakistan,* [2]*Department of Physics, Bacha Khan University, Charsadda, 24420, Pakistan,*

[3]*Department of Materials Science and Engineering, Le Quy Don Technical University, Hanoi 100000, Vietnam*

Supplementary Informations

Table SI: Lattice constant (a in Å), bond length (in Å), Work function (ϕ in eV) and potential (ΔV in eV) of CS-MX_2 (M=Mo, W; X=S, Se, Te) and CS-MXY ((X≠Y)=S,Se,Te) MS vdWH.

CS-MX_2	CS-MoS_2	CS-$MoSe_2$	CS-$MoTe_2$	CS-WS_2	CS-WSe_2	CS-WTe_2
ϕ	2.15	1.92	2.52	2.18	2.25	3.00
ΔV	-9.88	-13.78	-0.53	-9.88	-12.88	-1.84
m_h^*	0.025	0.092	0.074	0.039	0.071	0.102
m_e^*	0.019	0.039	0.051	0.027	0.051	0.082
CS-MXY(Model-I)	CS-MoSSe	CS-MoSTe	CS-MoSeTe	CS-WSSe	CS-WSTe	CS-WSeTe
ϕ	2.15	2.96	2.46	1.96	2.19	2.25
ΔV	-9.88	-9.63	-12.76	-10.14	-9.36	-11.68
m_h^*	0.058	0.087	0.064	0.052	0.086	0.074
m_e^*	0.024	0.050	0.038	0.029	0.047	0.061
CS-MXY(Model-II)	CS-MoSSe	CS-MoSTe	CS-MoSeTe	CS-WSSe	CS-WSTe	CS-WSeTe
ϕ	1.90	2.44	2.24	1.70	2.19	2.53
ΔV	-12.48	-0.24	-1.12	-13.26	-0.88	-1.05
m_h^*	0.079	0.13	0.087	0.103	0.073	0.118
m_e^*	0.037	0.09	0.054	0.079	0.051	0.064

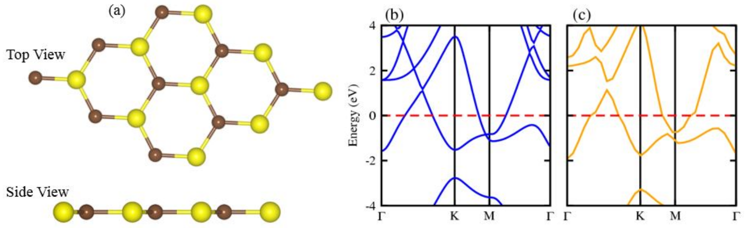

Figure S1. (a)Top and side view of the geometrical structure along with the band structure using (b)PBE, and (c)HSE06 functional for CS monolayer.

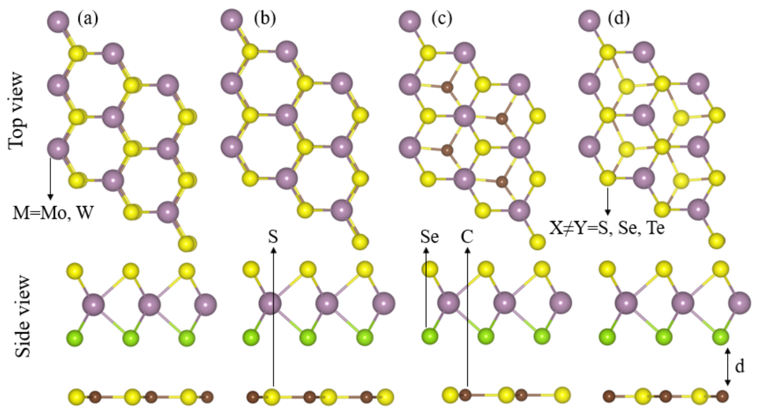

Figure S2. Possible stacking configurations of the ((a)-(d)) CS-MXY (M=Mo, W; X=S, Se, Te) MS vdWH in model-II, see text for details.

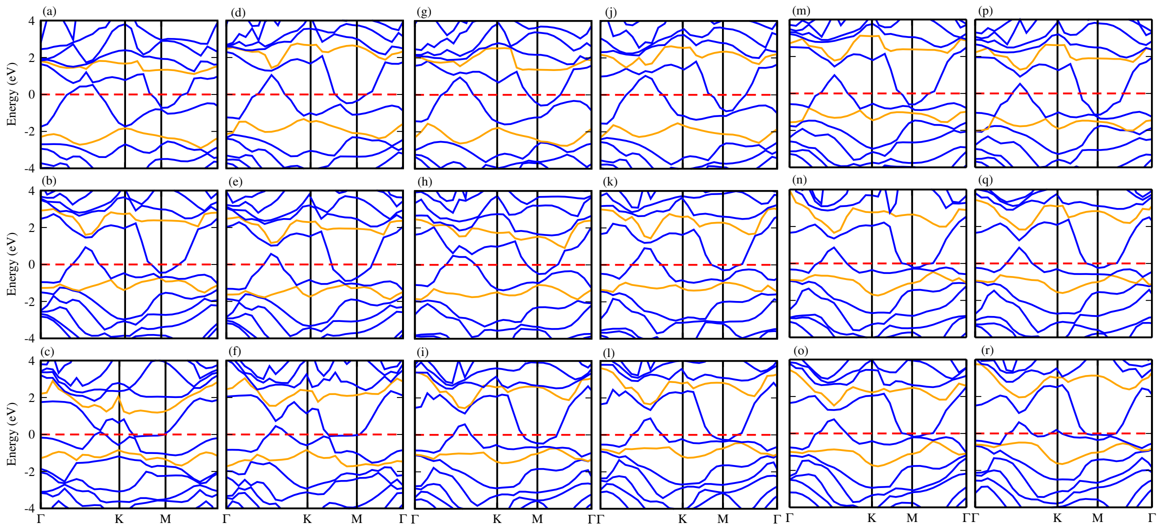

Figure S3. Band structures of (a) CS-MoS_2, (b)CS-$MoSe_2$, (c)CS-$MoTe_2$, (d) CS-WS_2, (e) CS-WSe_2, (f) CS-WTe_2, (g)CS-MoSSe, (h)CS-MoSTe (i) CS-MoSeTe, (j) CS-WSSe, (k) CS-WSTe, (l) CS-WSeTe, (m) CS-MoSeS (n) CS-MoTeS (o) CS-MoTeSe (p) CS-WSeS, (q) CS-WTeS (r) CS-WTeSe MS vdWH using HSE06 functionals.

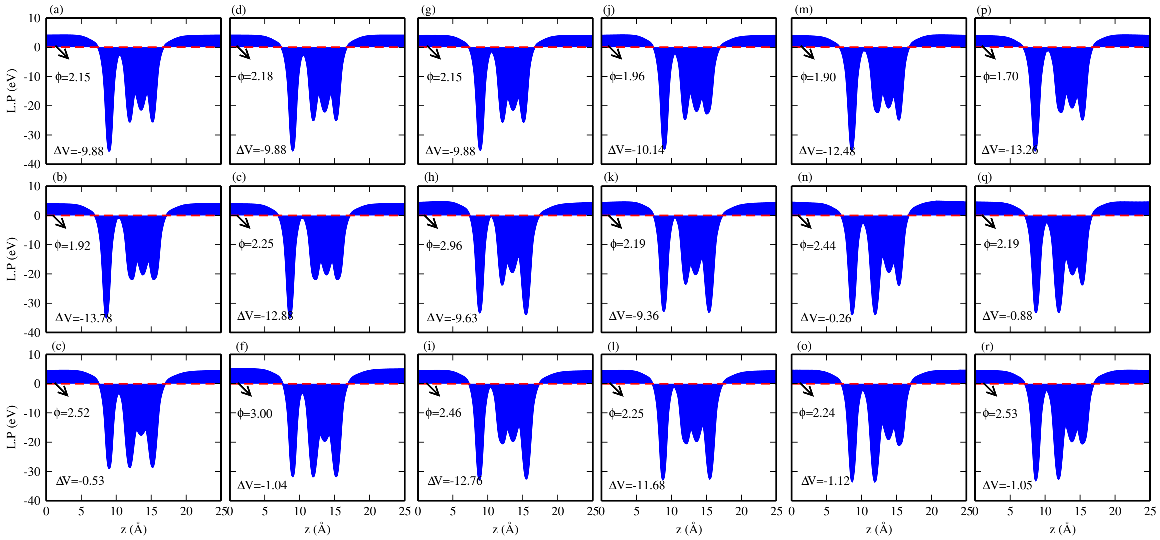

Figure S4. Average electrostatic potential of (a) CS-MoS_2, (b)CS-$MoSe_2$, (c)CS-$MoTe_2$, (d) CS-WS_2, (e) CS-WSe_2, (f) CS-WTe_2, (g)CS-MoSSe, (h)CS-MoSTe (i) CS-MoSeTe, (j) CS-WSSe, (k) CS-WSTe, (l) CS-WSeTe, (m) CS-MoSeS (n) CS-MoTeS (o) CS-MoTeSe (p) CS-WSeS, (q) CS-WTeS (r) CS-WTeSe MS vdWH using HSE06 functionals.

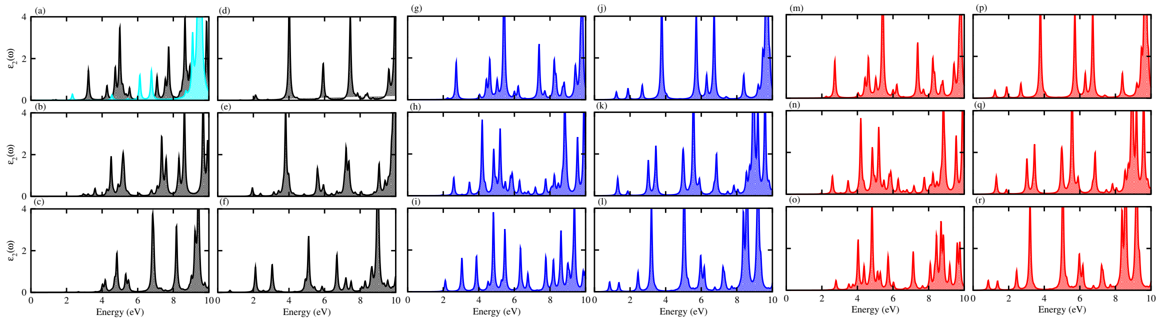

Figure S5. $\epsilon_2(\omega)$ of (a) MoS_2 (CS), (b) $MoSe_2$, (c) $MoTe_2$, (d) WS_2, (e) WSe_2, (f) WTe_2, (g) MoSSe, (h)MoSTe (i)MoSeTe, (j) WSSe, (k) WSTe, (l) WSeTe, (m) MoSeS (n) MoTeS (o) MoTeSe (p) WSeS, (q) WTeS (r) WTeSe monolayers.
